# Reappraising prediction of surgical complexity of non-functioning pituitary adenomas after transsphenoidal surgery: the modified TRANSSPHER grade

**DOI:** 10.1007/s11102-024-01495-9

**Published:** 2025-02-03

**Authors:** Giorgio Fiore, Giulio A. Bertani, Stephanie E. Baldeweg, Anouk Borg, Giorgio Conte, Neil Dorward, Emanuele Ferrante, Ziad Hussein, Anna Miserocchi, Katherine Miszkiel, Giovanna Mantovani, Marco Locatelli, Hani J. Marcus

**Affiliations:** 1 Unit of Neurosurgery, Fondazione IRCCS Ca’ Granda Ospedale Maggiore Policlinico, Milan, Italy; 2 Department of Neurosurgery, National Hospital for Neurology and Neurosurgery, London, UK; 3 Department of Pathophysiology and Transplantation, University of Milan, Milan, Italy; 4 Centre for Obesity & Metabolism, Department of Experimental & Translational Medicine, Division of Medicine, University College London, London, UK; 5 Unit of Neuroradiology, Fondazione IRCCS Ca’ Granda Ospedale Maggiore Policlinico, Milan, Italy; 6 Unit of Endocrinology, Fondazione IRCCS Ca’ Granda Ospedale Maggiore Policlinico, Milan, Italy; 7 Department of Neuroradiology, National Hospital for Neurology and Neurosurgery, London, UK; 8 Department of Clinical Sciences and Community Health, University of Milan, Milan, Italy

**Keywords:** Adenomas, Gross-total resection, Machine learning, Neuroendocrine tumor, PitNET, Pituitary

## Abstract

**Purpose:**

Prognostication of surgical complexity is crucial for optimizing decision-making and patient counseling in pituitary surgery. This study aimed to develop a clinical score to predict gross-total resection (GTR) in non-functioning pituitary adenomas (NFPAs) using externally validated machine-learning (ML) models.

**Methods:**

Clinical and radiological data were collected from two tertiary medical centers. Patients had pre- and postoperative structural T1-weighted MRI with gadolinium and T2-weighted preoperative scans. Three ML classifiers were trained on the National Hospital for Neurology and Neurosurgery dataset and tested on the Foundation IRCCS Ca’ Granda Polyclinic of Milan dataset. Feature importance analyses and hierarchical-tree inspection identified predictors of surgical complexity, which were used to create the grading score. The prognostic performance of the proposed score was compared to that of the state-of-the art TRANSSPHER grade in the external dataset. Surgical morbidity was also analyzed.

**Results:**

All ML models accurately predicted GTR, with the random forest classifier achieving the best performance (weighted-F1 score of 0.87; CIs: 0.71, 0.97). Key predictors—Knosp grade, tumor maximum diameter, consistency, and supra-sellar nodular extension—were included in the modified (m)-TRANSSPHER grade. The ROC analysis showed superior performance of the m-TRANSSPHER grade over the TRANSSPHER grade for predicting GTR in NFPAs (AUC 0.85 vs. 0.79).

**Conclusions:**

This international multi-center study used validated ML algorithms to refine predictors of surgical complexity in NFPAs, yielding the m-TRANSSPHER grade, which demonstrated enhanced prognostic accuracy for surgical complexity prediction compared to existing scales.

## Introduction

Non-functioning pituitary adenomas (NFPAs) are clonal neoplastic proliferations of the anterior pituitary hormone-producing cells [1], characterized by the absence of clinical evidence of hormonal hypersecretion [2]. Recently, the term pituitary neuroendocrine tumors (PitNETs) has been proposed in place of pituitary adenomas [1]. PitNets are characterized by a great heterogeneity in their clinical behavior, histopathological features, and invasion into surrounding structures [3], with non-functioning neoplasms having a higher incidence of invasion than functioning tumors [1]. Growing toward the cavernous sinus (CS) and the suprasellar cisterns, NFPAs may compress the surrounding neurovascular and endocrine structures, eventually causing visual defects and hypopituitarism [1, 4–8]. Operative treatment is usually indicated to alleviate mass effect symptoms or prevent their onset in case of significant tumor growth [3].

The surgical outcome of NFPAs depends on several anatomical, tumor, and surgeon-related factors that make the prediction of surgical complexity challenging. Key variables influencing gross-total resection (GTR) include tumor size, shape, invasiveness, and consistency [9, 10]. These variables, and their effects on tumor resection, are often interlinked, making any statistical model that captures these often non-linear relationships complex [11]. Machine learning (ML) offers a promising approach to addressing this complexity [11–15]. Leveraging the predictive power of externally validated ML models, this study aimed to build a clinical score to anticipate the surgical complexity of NFPAs by predicting GTR using radiological preoperative measures.

## Methods

### Study design

This study is the result of an international collaboration between the National Hospital for Neurology and Neurosurgery (NHNN, London, UK) and the Fondazione IRCCS Ca’ Granda Ospedale Maggiore—Polyclinic of Milan (PM, Milan, Italy). The data included in this study were extracted from prospectively maintained databases of patients undergoing pituitary surgery at the two institutions. The NHNN dataset was used to develop the machine learning model and clinical score, and the PM dataset was used to provide external validation. All procedures performed in studies involving human participants were in accordance with the ethical standards of the institutional and/or national research committee and with the 1964 Helsinki Declaration and its later amendments or comparable ethical standards. Patient inclusion was approved by the ethical committees of Milan (n. 747_2017) and Westminster Research Ethics (IRAS protocol code 251,432).

### Variables and outcome of interest

To enhance the clinical utility of the current investigation, GTR was chosen as the measure of surgical complexity [10]. For a scan to be scored as GTR, the absence of residual tumor on at least two consecutive thin-cut MRI slices had to be confirmed [10]. The selection of the predictive variables was based on the literature focusing on this topic [9, 10]. The inter-carotid distance was the main anatomical variable of interest; while tumor maximum diameter (on any plane) [10], Knosp grade [16], and sellar, para-sellar and supra-sellar tumor invasion according to the Hardy-Wilson classification [17], were identified as the main morphological tumor variables of interest. Tumor texture was assessed using the T2 signal intensity ratio (T2SIR) which involves measures of tumor and cerebrospinal fluid (CSF) signal intensity, and it was proven to be a reliable marker of NFPA consistency, unlike previously proposed T2-based indicators [9]. The T2SIR was calculated using the published formula: T2SIR = [(T2 tumor mean SI–SD)/T2 CSF SI], where SD represents the standard deviation of the T2 tumor signal intensity.

### Data collection

Experienced neuroradiologists (G.C. and K.M.) at the two institutions reviewed the preoperative and postoperative images to assess GTR and were blinded to the measured variables of interest. Experienced neurosurgeons (G.F., H.J.M., and G.A.B.) measured the variables of interest on standard workstations and were blinded to the GTR.

### Inclusion criteria

Patients who underwent trans-nasal-sphenoidal (TNS) surgical resection of NFPAs at the two institutions were enrolled in the present study if they had preoperative and postoperative (within 3–6 months) structural 3D T1-weighted (w) MRI images before and after gadolinium administration and preoperative T2w scans.

Tumors with a large (more than 50%) necrotic or cystic component had a high risk of potentially biasing the results of this investigation, both in terms of predicted outcome (GTR or STR) and of preoperative characterization (tumor consistency and infiltration into surrounding compartments). Therefore, these tumors were excluded from the analysis.

### Surgical morbidity

An analysis of surgical morbidity was conducted, including data on the occurrence of CSF leaks, visual worsening, carotid injury, and pituitary hematoma after surgery. Also, endocrinological data were collected through extensive preoperative and postoperative pituitary assessments. Pituitary deficiencies were defined according to Hussein et al. [18] for NHNN and Fleseriu et al.[19] for PM. Postoperative pituitary deficits were classified as definitive if confirmed one year after surgery, with rates and types of hormone deficits reported. Additionally, data related to visual assessments before and after surgery were provided.

### Statistical analysis

Frequencies were reported as percentages and compared by chi-squared and Fisher exact tests according to sample size. The continuous normally distributed variables were reported as mean ± standard deviation and compared with Student’s t-test or variance analyses. The continuous skewed distributed variables were reported as median (interquartile range, IQR) and compared via Mann–Whitney U-test and Kruskal–Wallis test. To ensure consistency with previous reports, logistic regression was employed to assess the linear relationship between the explanatory variables and the main outcome in the NHNN model developing population. Statistical significance was reached for p-values inferior to 0.05. All statistical analyses were performed using IBM SPSS (version 28.0, International Business Machines Corp, New York, USA) and R software 4.2.2 (R Foundation for Statistical Computing, Vienna, Austria; http://www.r-project.org/index.html).

### Machine learning

A random forest tree (RFT), a decision tree (DT), and a logistic regression (LR) classifier were trained and optimized to predict GTR in NFPAs. The ML algorithms were initiated using the sci-kit learn library on Python v.3.9. Tuning of hyperparameters was obtained using GridSearch fivefold cross-validation on the (NHNN) model-developing cohort. The hyperparameters achieving the highest weighted F1-score were exported and included in the classifier. For each model, the classifier performance (internal validation) was taken as the average weighted F1-score over the cross-validation folds along with 95% confidence intervals. Further, the apparent performance on the training data was reported. For patients in the PM external validation cohort, GTR prediction was performed by each optimized classifier and the weighted F1-score was computed. The 95% CIs were calculated by bootstrapping the point estimates through random resampling with replacement. There was no need for recursive feature elimination as only a limited number of variables were purposefully used. Machine learning analyses were run on a Jupyter Notebook using Pandas, Numpy, Matplotlib, Seaborn, and Sklearn on Python v.3.9. This study followed the TRIPOD (Transparent Reporting of a multivariable prediction model for Individual Prognosis or Diagnosis) guidelines [20, 21].

### Clinical score

For each algorithm, a feature importance analysis was conducted. The single tree generated by the DT classifier was printed and inspected to thoroughly understand the relationship between the predictive variables and GTR. Based on the feature importance analysis and DT nodes, a grading score was developed to anticipate surgical complexity. The prognostic performance of the proposed grading score to predict GTR of NFPAs was evaluate alongside that of a state-of-the-art scale, the TRANSSPHER grade [10], by measuring the area under the receiver operating characteristic curve (ROC-AUC) on the validation cohort. The predictive performances of the two grading scores were further compared using the Akaike Information Criterion (AIC), a widely recognized method for comparing predictive models. AIC assesses the goodness of fit while penalizing model complexity to prevent overfitting. By balancing these factors, AIC identifies models that are both accurate and generalizable, making it particularly suited for clinical applications [22, 23]. Lower AIC values indicate a model with superior performance.

### Data availability

The code and anonymized data supporting the findings of this study are available from the corresponding author upon reasonable request.

## Results

The model-developing cohort constituted of 209 patients receiving TNS NFPA surgical resection at NHNN between February 2005 and November 2017. The external validation cohort comprised 37 patients undergoing the same surgical procedure at PM between April 2018 and November 2022. Patient demographics and clinical features of the model-developing cohort and external validation cohort are summarized in Tables 1 and 2.

**Table 1:** Patient demographics and clinical features of the model developing cohort

Variable	GTR	STR	P-value
N	95	114	
Age	57 ± 14	60 ± 12	0.11*
Intercarotid Distance	18.7 ± 4.6	21.4 ± 5	< 0.001*
T2SIR	0.55 ± 0.1	0.48 ± 0.1	< 0.001*
Tumor maximum diameter	23.9 ± 6.8	30.1 ± 8.2	< 0.001*
Hardy-Wilson Sellar 1 2 3 4	13/95 (13.7%) 37/95 (38.9%) 39/95 (41.1%) 6/95 (6.3%)	6/114 (5.3%) 24/114 (21.1%) 55/114 (48.2%) 29/114 (25.4%)	< 0.001**
Hardy-Wilson Suprasellar A B C D/E	46/95 (48.4%) 42/95 (44.2%) 7/95 (7.4%) 0	38/114 (33.3%) 58/114 (50.9%) 15/114 (13.2%) 3/114 (2.6%)	0.05**
Knosp Grade 1 2 3 4	44/95 (46.3%) 40/95 (42.1%) 8/95 (8.5%) 3/95 (3.2%)	23/114 (20.2%) 32/114 (28.1%) 27/114 (23.7%) 32/114 (28%)	< 0.001**

^*^denotes t-test; **denotes chi-squared test

**Table 2:** Patient demographics and clinical features of the external validation cohort

Variable	GTR	STR	P-value
N	17	20	
Age	63 ± 13	59 ± 16	0.45*
Intercarotid Distance	20.5 ± 3.8	30.7 ± 9	0.35*
T2SIR	0.63 ± 0.1	0.5 ± 0.1	0.003*
Tumor maximum diameter	20.5 ± 3.8	30.7 ± 9.1	< 0.001*
Hardy-Wilson Sellar 1 2 3 4	4/17 (23.5%) 13/17 (76.5%) 0 0	0 13/20 (65%) 5/20 (25%) 2/20 (10%)	0.01**
Hardy-Wilson Suprasellar A B C D/E	15/17 (75%) 2/17 (11.8%) 0 0	6/20 (30%) 10/20 (50%) 3/20 (15%) 1/20 (5%)	0.004**
Knosp Grade 1 2 3 4	7/17 (41.2%) 7/17 (41.2%) 3/17 (17.6%) 0	0 7/20 (35%) 10/20 (50%) 3/20 (15%)	0.003**

^*^denotes t-test; **denotes chi-squared test

### Predictors’ dispersion

Tumor consistency, Knosp grade, tumor maximum diameter, intercarotid distance, sellar, para-sellar and supra-sellar tumor invasion were all significantly related to GTR in the univariate analysis in the model-developing cohort (Table 1). The multivariate logistic regression model (R = 0.6, p < 0.001) identified the following variables as independently associated with GTR: Knosp grade (b = − 0.66, p < 0.001), tumor consistency (b = 5.77, p < 0.001), and tumor maximum diameter (b = − 0.09, p = 0.035).

### Machine learning

**Fig. 1:**
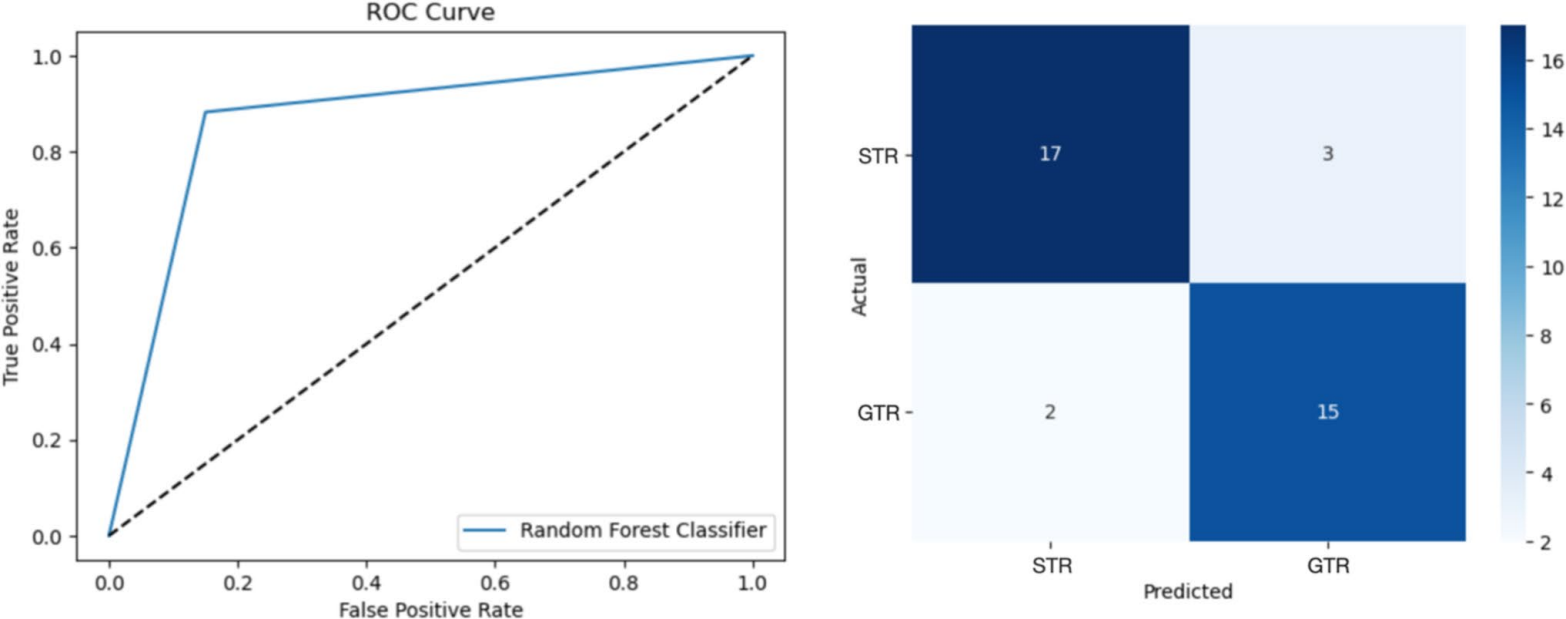
ROC-AUC and Confusion matrix of RFT classifier performance on the external validation cohort. The figure shows the ROC curve for the RFT algorithm to predict GTR in NFPAs along with the confusion matrix of actual and predicted resection outcomes in the external validation cohort

The RFT algorithm was the best classifier with a weighted F1-score of 0.74 ± 0.05 and an apparent performance of a weighted F1 equal to 0.8 in the model-developing cohort, and a weighted F1 score in the external validation cohort of 0.87 (CIs: 0.71; 0.97; Table 3). Out of 20 patients in the external validation cohort receiving STR, the RFT correctly classified 17 patients. Further, the algorithm correctly classified 15 out of 17 patients undergoing GTR. The overall estimated ROC-AUC was 0.87 (Fig. 1). The confusion matrix is available in Fig. 1. The most important features used by the RFT algorithm to predict GTR were, in decreasing order: tumor maximum diameter, tumor consistency, and Knosp grade.

**Table 3:** Machine-Learning Prognostic Performances

Algorithm	Model-Developing Cohort		Test Cohort
	Internal Validation	Apparent Performance	External Validation
RFT			
*F1-score*	0.74 ± 0.05	0.8	0.87 (0.71; 0.97)
DT			
*F1-score*	0.68 ± 0.05	0.78	0.78 (0.55; 0.9)
LR			
*F1-score*	0.69 ± 1	0.71	0.81 (0.6; 0.92)

*DT* decision tree; *LR* logistic regression; *RFT* random forest tree

**Fig. 2:**
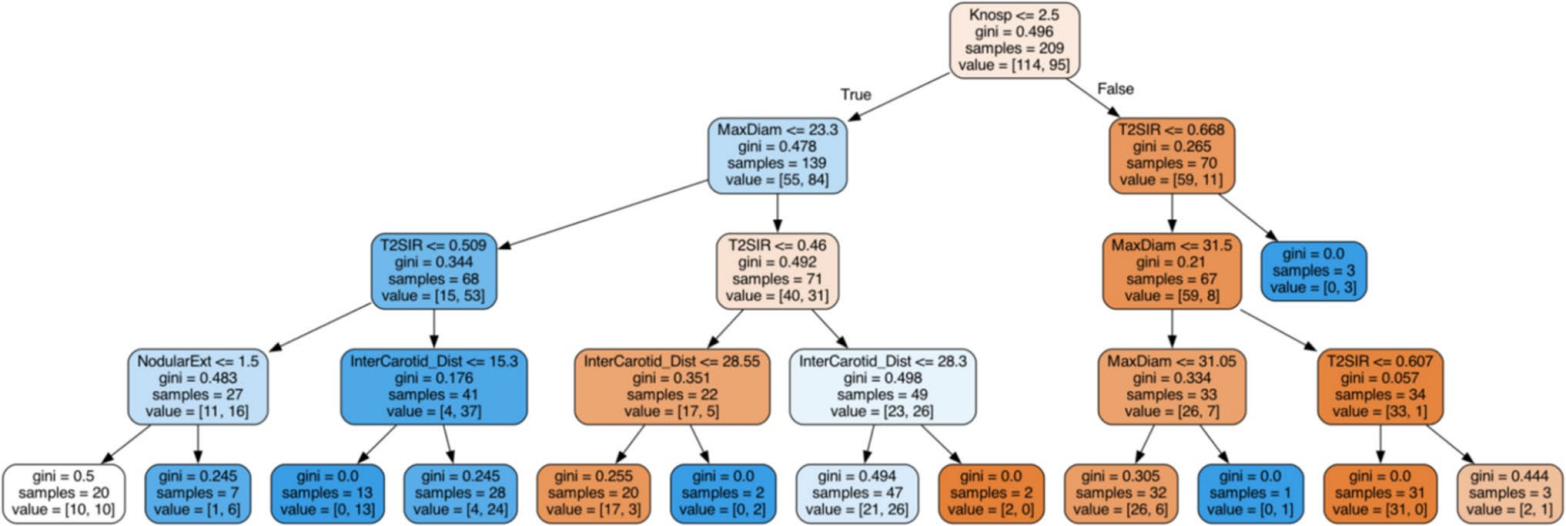
Hierarchical Tree employed by the DT classifier. For tumors infiltrating the cavernous sinus (Knosp grade 3 or greater), the chance of achieving a GTR dropped to 12% for the whole series. Among them, one third of NFPAs showed very soft consistency (T2SIR greater than 0.6) and two thirds had a maximum diameter less than 31 mm. For non-infiltrating NFPAs, having a maximum diameter greater than 23 mm meant to reduce the chance of GTR to 37%, which further dropped to 5% if the tumors showed a hard consistency (T2SIR less than 0.4). For non-invasive and small (tumor maximum diameter less than 23 mm) NFPAs, an intermediate to hard consistency (T2SIR less than 0.5) reduced the rate of GTR to 30%, which dropped to 11% if the tumor also presented with a nodular supra-sellar extension. On the other hand, for small tumors showing an intermediate to soft consistency (T2SIR greater than 0.5) the chance to achieve a GTR was of 70% which dropped to 25% in case of a very tight surgical corridor with an inter-carotid distance less than 15 mm. For large tumors, increasing values of inter-carotid distance were related to reduced rates of GTR

The LR and DT models showed comparable classification performances (Table 3). The overall estimated ROC-AUC of LR was 0.81, and the most important features were: tumor consistency, the Knosp grade, and the tumor suprasellar extension. The overall estimated ROC-AUC of DT was 0.78, and the most important features used by the algorithm were: the Knosp grade, tumor consistency, and tumor maximum diameter. The hierarchical tree is available in Fig. 2.

### The modified(m)-TRANSSPHER grade

**Fig. 3:**
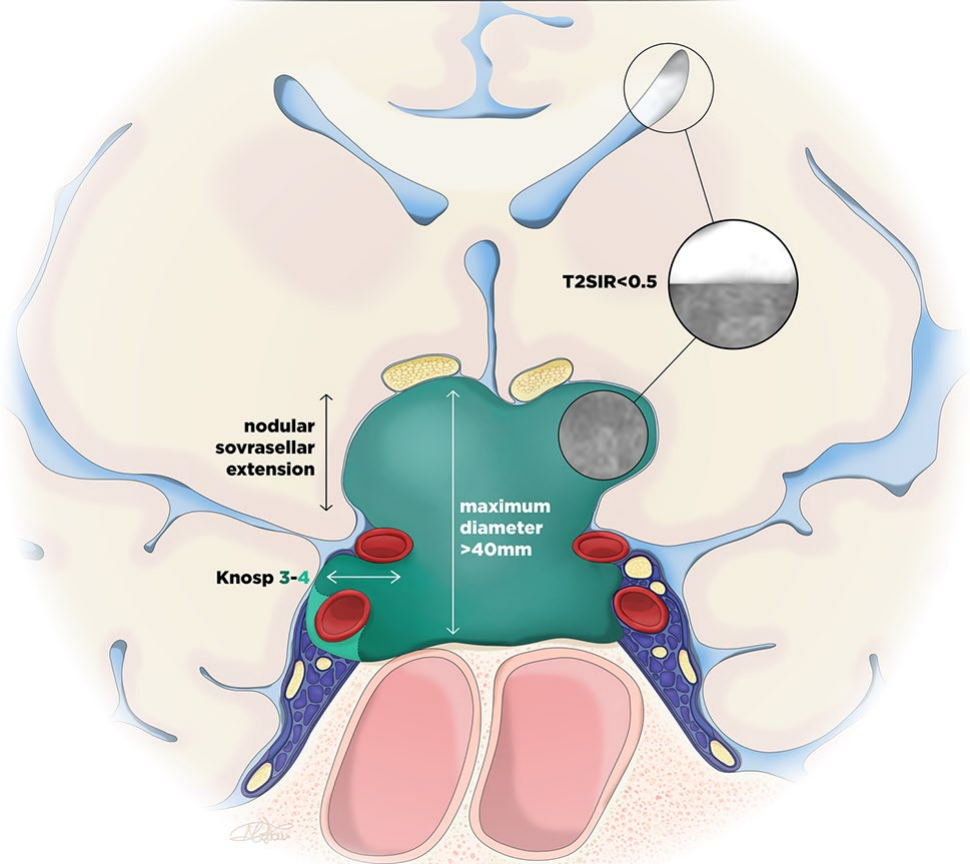
The modified TRANSSPHER grade. A schematic illustration showing the modified TRANSSPHER grade. One point is assigned for tumor diameter superior to 40 mm, Knosp grade 3 or greater, tumor supra-sellar nodular extension, and T2SIR inferior to 0.5

The feature importance analyses and the hierarchical tree inspection indicated the Knosp grade, tumor maximum diameter, tumor consistency, and tumor supra-sellar nodular extension as the most reliable predictors of GTR. The already validated TRANSSPHER grade included all these variables except for tumor consistency. Thus, a modified TRANSSPHER (m-TRANSSPHER) grade was proposed. As the TRANSSPHER grade, the m-TRANSSPHER grade assigned one point for tumor diameter superior to 40 mm, Knosp grade 3 or greater, and tumor supra-sellar nodular extension. Further, the m-TRANSSPHER grade assigned an additional point for a T2SIR inferior to 0.5 (intermediate to hard tumor consistency). An illustration showing the m-TRANSSPHER grade is available in Fig. 3. The higher the m-TRANSSPHER grade the lower the chances of GTR.

**Fig. 4:**
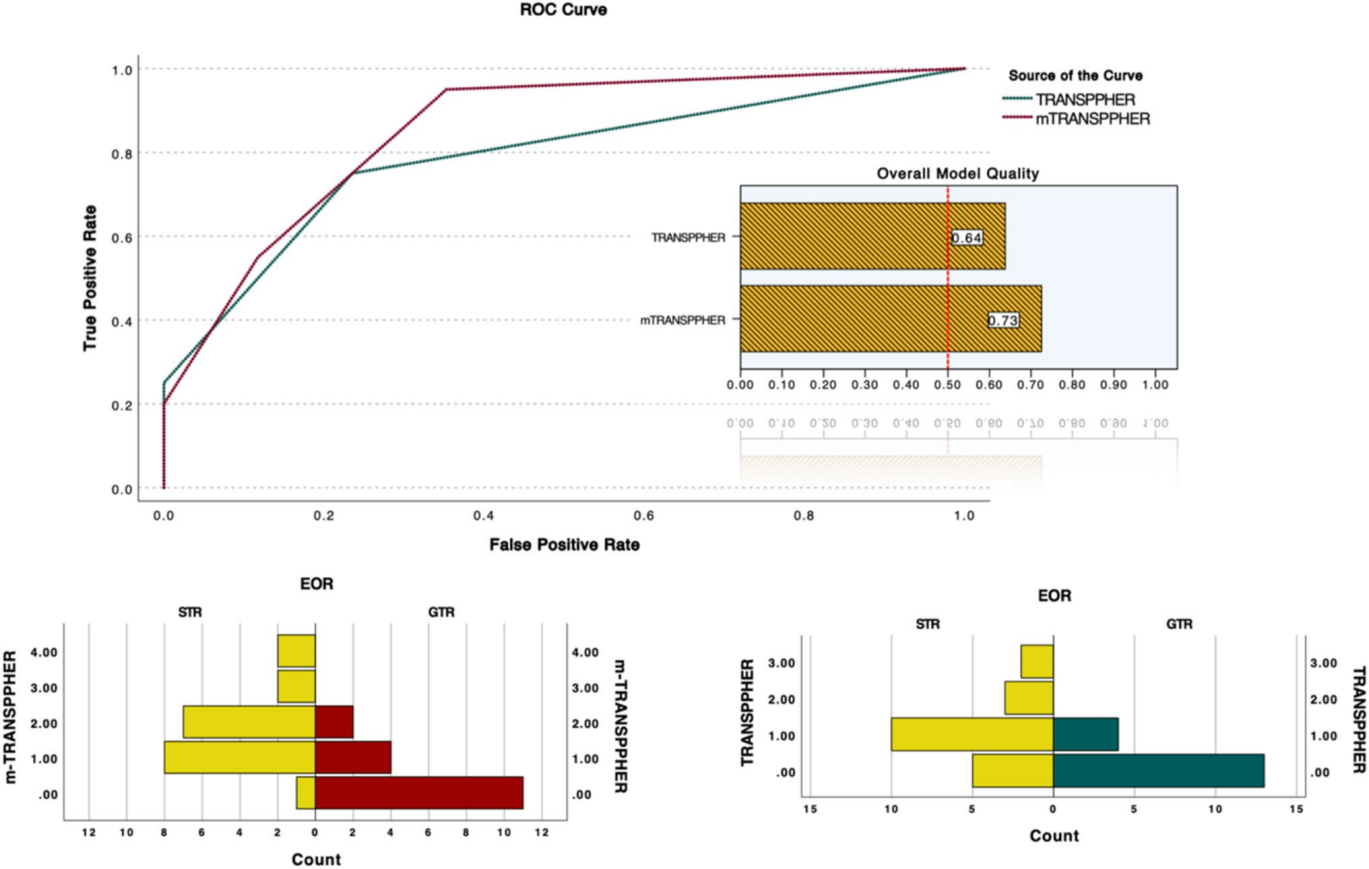
ROC curve analysis and population pyramid distribution of GTR for the TRANSSPHER and m-TRANSSPHER grades. The ROC analysis shows a better prognostic performance for the m-TRANSSPHER grade compared to the TRANSSPHER grade to predict GTR in NFPAs, with an overall model quality of 0.73 for the m-TRANSSPHER grade and 0.64 for the TRANSSPHER grade. The population pyramid distribution of GTR demonstrates a better dispersion of the outcome prognostications among the classes of the m-TRANSSPHER grade compared to those of the TRANSSPHER grade

The m-TRANSSPHER grade demonstrated refined prognostic performance for predicting GTR in NFPAs compared to the TRANSSPHER grade, with lower AIC value (m-TRANSSPHER: 37.9, TRANSSPHER: 42.4) and residual deviance (m-TRANSSPHER: 33.9, TRANSSPHER: 38.4). Additionally, the m-TRANSSPHER grade showed a higher AUC (0.85) compared to the TRANSSPHER grade (0.79, Fig. 4).

### Surgical morbidity

Three patients in the model-developing cohort and one patient in the model-validation cohort experienced postoperative CSF leaks, leading to an overall rate of 1.4% and 2.7%. Two patients in the model-developing cohort experienced a pituitary hematoma and a carotid injury, respectively (overall incidence of 0.5%). No other surgical complications were reported. One hundred and thirty-nine patients (66.5%) in the model-developing cohort experienced a postoperative visual improvement, while only three (1.4%) patients retained their preoperative visual deficit. Sixty-six patients (31.6%) had a normal preoperative visual assessment. Similarly, 23 patients (62.2%) in the external validation cohort had an improved visual outcome after surgery, with one patient (2.7%) retaining the preoperative visual deficit. Thirteen patients (35.1%) had a normal preoperative visual assessment. No patients in the two cohorts experienced visual worsening after surgery.

For patients in the model-developing cohort, 79.9% showed a normal postoperative anterior pituitary function. When a new anterior pituitary hormone deficit was found, patients were more likely to experience a secondary hypocortisolism (7.7%). Seventeen patients developed permanent diabetes insipidus (8.1%).

For patients in the validation cohort, 78.4% showed a normal postoperative anterior pituitary function. After surgery, the most frequently diagnosed anterior pituitary deficit was a secondary hypocortisolism (8.1%). Three patients (8.1%) experienced a permanent diabetes insipidus. Data regarding endocrinological outcomes in the two cohorts are summarized in Table 4.

**Table 4:** Endocrinological Outcomes

Hormone Deficiency	Model-Developing Cohort	Test Cohort
Anterior Pituitary		
*No Deficit*	167 (79.9)	29 (78.4)
*GH*	1 (0.5)	0
*FSH/LH*	3 (1.4)	0
*ACTH*	16 (7.7)	3 (8.1)
*TSH*	2 (1)	1 (2.7)
*Hypopituitarism*	20 (9.5)	3 (8.1)
DI	17 (8.1)	3 (8.1)

Values are presented as number of patients (%)

*ACTH* adrenocorticotropic Hormone; *DI* diabetes insipidus; *FSH/LH* follicle-stimulating hormone (FSH)/luteinizing hormone (LH); *GH* growth Hormone; *TSH* thyroid-stimulating hormone

No correlations were found between data of surgical morbidity and surgical complexity measured through the m-TRANSSPHER score as well as between surgical morbidity and classes of surgical resection (see Table 5).

**Table 5:** Surgical Morbidity, m-TRANSSPHER grade, and Resection Outcomes

Test Variable	Model-Developing Cohort		P-value	Test Cohort		P-value
	mT grade < 2	mT grade > 2		mT grade < 2	mT grade > 2	
Surgical Complication			0.254*			1*
No	165	39		23	13	
Yes	3	2		1	0	
New Hormone Deficit			0.084			1*
No	116	34		17	9	
Yes	52	7		7	4	
	GTR	STR		GTR	STR	
Surgical Complication			0.379*			1*
No	94	110		17	19	
Yes	1	4		0	1	
New Hormone Deficit			0.878			0.279*
No	69	81		10	16	
Yes	26	33		7	4	

Values are presented as number of patients. mT: m-TRANSSPHER; GTR: gross-total resection; STR: sub-total resection

^*^denotes Fisher’s Exact Test; **denotes Pearson Chi-Square

## Discussion

### Principal findings

In this international study, ML-based analyses were used to develop the modified TRANSSPHER grade. The score showed a refined ability to predict surgical complexity compared to the state-of-the-art TRANSSPHER grade, as shown by ROC analyses and AIC comparisons. These refinements build on the strengths of the original scale by retaining key features such as tumor size, suprasellar extension, and cavernous sinus infiltration, while incorporating radiological measures of tumor consistency. A better prognostication of surgical complexity may have significant implications. The simplicity and rapid calculation of the m-TRANSSPHER grade make it a valuable tool for preoperative counseling, enabling patients to be informed about the surgical goal and the potential need for closer postoperative monitoring. Additionally, the score may support multidisciplinary pituitary board discussions to better stratify expected outcomes. This could be especially relevant for frail patients, where a careful evaluation of the risk–benefit trade-off is essential to guide management decisions. Furthermore, the score may contribute to standardizing the reporting of surgical complexity in future studies on NFPAs.

The ML algorithms employed to develop the m-TRANSSPHER grade showed robust performances. Particularly, the RFT classifier was able to predict GTR in NFPAs with high precision and sensitivity (weighted F1 score of 0.74 and 0.87 in the development and external validation cohorts, respectively). Predictive models generally outperform explanatory or descriptive statistics in accurately quantifying the predictability of measurable phenomena [14, 24]. Therefore, predictive models may provide a more accurate estimate of the possibility of predicting a certain phenomenon, such as tumor GTR [14]. The high accuracy of the hereby proposed ML models to correctly classify GTR in NFPAs yields a new, more reliable [12, 24] and strong “reality check[14]” of the predictive value of the aforementioned variables and about the effectivness of preoperative tools for assessing surgical complexity in pituitary surgery.

### Predictors of surgical complexity in NFPAs

Invasion of the cavernous sinus deeply affects pituitary surgical complexity as infiltrative pituitary adenomas tend to invade not only the medial wall of the cavernous sinus [16, 25], whose initial tumor infiltration still remains surgically amenable in experienced hands [26] but also other regions of the cavernous sinus dural enclosure, the internal carotid artery adventitia, and the cranial nerves [27]. Despite discriminating cavernous sinus invasion from compression might be challenging on preoperative MRI [28], the preoperative Knosp classification has been widely validated as a predictor of tumor resection in pituitary surgery [7, 9, 10, 16, 25, 29, 30]. The results of our predictive models are in line with the previous literature, with invasive NFPAs (tumor Knosp grade greater than 2) being strongly associated with reduced chances of GTR.

The reliability of tumor maximum diameter to prognosticate EOR in NFPAs has been largely described [10, 29, 31]. Nonetheless, the debate about the best method to assess tumor size when predicting tumor resection is still an object of open debate. Volumetric measures have been claimed to be more reliable than bi-dimensional measurements since they consider all three planes in which cross-sectional length can be measured [32]. On the other hand, tumor maximum diameter in any plane represents a simple and reproducible way to assess tumor size, with the advantage of being less time-consuming compared to tumor volume. Thus, the inclusion of this measure fits well with the objective of this study: that is, to translate the results of complex ML algorithms into a simple tool to predict surgical complexity. Further, the concurrent inclusion of measures of antero-posterior, cranio-caudal and transverse tumor invasion should limit the drawbacks of using a bi-dimensional measure of tumor size. In line with this hypothesis, Mooney et al. [10] already found no significant differences between the predictive value of tumor diameter and tumor volume when predicting GTR in NFPAs along with measures of para-sellar tumor invasion. We used a cut-off of 40 mm as it is still widely recognized as pivotal to distinguish giant pituitary adenomas from their counterpart [5].

Even though the importance of tumor consistency for pituitary surgery has been documented for a long time [33], its prediction using simple T2w images has been challenging for a while [34]. Recently, a new parameter of tumor consistency (the T2SIR) has been proposed [9]. Overcoming the limitations of early methods, the T2SIR was built with high attention to the standardization and heterogeneity of signal intensity measurements of NFPAs. Thus, it proved to be a reliable predictor of NFPAs’ collagen content, wigh high sensitivity and specificity [9]. Further, the T2SIR was strongly associated with tumor extent of resection; firm tumors showed reduced T2SIR values and were less likely to receive GTR [9]. The results hereby reported replicated those findings. The hierarchical tree showed that almost 40% of invasive NFPAs, which by definition are unlikely to receive GTR, are still completely resectable if they have soft consistency (T2SIR greater than 0.6). On the other hand, non-invasive tumors offer additional challenges to undergo GTR when they present with an intermediate-to-hard consistency (T2SIR less than 0.5). Tumor consistency was the predictive variable most exploited by the employed ML algorithms, confirming the importance of taking into account tumor texture to predict surgical complexity in NFPA trans-sphenoidal surgery.

The fourth feature composing the m-TRANSSPHER grade was the presence of a suprasellar tumor invasion. Again, the importance of this variable may be explained by inspecting the hierarchical tree. A subgroup of tumors that are not invasive (tumor Knosp grade less than 3) struggle to receive GTR when they show an intermediate-to-hard consistency and they grow in the suprasellar cistern toward the third ventricle or the frontal and temporal lobes (Hardy-Williams grade C-E). These tumors represents a real challenge for pituitary surgeons.

The inter-carotid distance was not included in the m-TRANSSPHER grade as it proved to be an unreliable predictor of NFPA surgical complexity. For tumors with a maximum diameter greater than 23 mm, increasing values of inter-carotid distance were related to reduced rates of GTR. On the contrary, for tumors with a maximum diameter of less than 23 mm, increasing values of inter-carotid distance were related to increased rates of GTR. The first counterintuitive result, which was in line with previous reports [10], is to be imputed to the internal carotid arteries’ displacement caused by large NFPAs. Therefore, increasing values of inter-carotid distance as a result of tumor growth relate to tumors of larger size that in turn are associated with reduced rates of GTR. Medium-to-small NFPAs do usually not displace the surrounding structures being confined in the sella turcica. In these cases, a wider surgical corridor related to greater rates of GTR (see the hierarchical tree in Fig. 2).

Finally, none of the employed ML models identified the rate of sellar floor tumor invasion as an important predictor of surgical complexity. This finding suggests that tumor infiltration toward the sphenoidal sinus or even downward is not as challenging as tumor invasion of the cavernous sinus or supra-sellar regions. Indeed, large tumors can still be amenable to GTR when they principally extend to the sphenoidal sinus or downward.

### Uncovering the relationship between predictors of surgical complexity in NFPAs

The RFT classifier outperformed the other implemented algorithms, namely LR and DT models. The implications coming from this finding are twofold. Firstly, the relationship between predictors of surgical complexity in NFPAs is more complex than what linear models and classical statistics can describe. Tumor soft consistency is important per se to achieve GTR. Nonetheless, this can be even more significant for tumors infiltrating the cavernous sinus and/or for big tumors with supra-sellar extension. Secondly, the relationship between the aforementioned predictors is not hierarchical. The DT model, which uses a hierarchical tree structure, identified the Knosp grade as the most important feature to classify surgical complexity. This result aligns with the previous literature and also holds for the majority of patients included in this study. However, the RFT model showed increased classification performances by adopting a non-hierarchical, random split of a subset of predictors each time. In a clinical scenario, non-infiltrating Knosp grade 2 NFPAs with large size extending to the supra-sellar compartments and/or with hard consistency may offer additional surgical challenges compared to infiltrating Knosp grade 3 NFPAs with soft consistency, reduced size, and/or absent nodular supra-sellar extension.

### Limitations and future prospects

A strength of this externally validated international ML-based study is that high predictive power did not come at the cost of interpretability. Rather, it was used to build a powerful simple prognostic scale of surgical complexity, and new insights into the relationship between predictors of GTR in NFPAs, and their association with the outcome variable, were described. However, the following limitations must be noted. Although we adhered to the TRIPOD guidelines for external validation—ensuring both geographical and temporal validation [20]— the relatively small cohort size may limit the generalizability of our findings, despite the model demonstrating minimal evidence of overfitting. Expanding validation to larger, multicenter cohorts will enhance the model’s applicability and robustness. Another limitation was the use of T2SIR as an indirect measure of tumor consistency. While T2SIR has been validated as a predictor of collagen content in NFPAs, direct assessments of tumor consistency are only feasible intraoperatively. Even if our findings provide further validation of T2SIR as an indicator of tumor consistency, future studies will explore the correlation between T2SIR and intraoperative consistency to strengthen its applicability. Additionally, we limited predictive features to six variables to enhance interpretability and reduce the risk of overfitting, potentially affecting predictive power [12, 35]. The algorithm’s performance on external validation supports this trade-off. Finally, because of the small numbers we cannot comment strongly on the distribution of surgical complications across the different grading classes of the score. Future investigations would be valuable.

## Conclusions

In this international multi-center study, externally-validated machine learning algorithms were used to investigate predictors of surgical complexity in NFPAs, delineating a better-defined clinical picture of variables affecting GTR. Their results were translated into the m-TRANSSPHER grade which produced a refined surgical complexity stratification when compared to state-of-the-art grading scales.
